# Proceedings of the Association of the Colleges of Dentistry

**Published:** 1868-05

**Authors:** J. Taft


### Proceedings of Societies.

### PROCEEDINGS OF THE ASSOCIATION OF THE COLLEGES OF DENTISTRY.

The Association met according to previous notice, in the Lecture room of the New York Dental College, Thursday, April 2d, 1868, at 11 A. M. President E. Parmley, in the chair.

The Colleges were represented as follows, viz.:

*Baltimore Dental College*—Profs. P. H. Austen, F. J. S. Gorgas.
*New York College of Dentistry*—Profs. E. Parmley, R. King Browne, N. W. Kingsley.
*Missouri Dental Colleye*—Prof. H. E. Peebles.
*Ohio College of Dental Surgery*—Prof. J. Taft.

The minutes of the last annual meeting were read.

It was suggested that reports be heard from each of the Colleges, more particularly with rcference to the influence of the Regulations adopted by this Association at its last meeting.

Prof. Gorgas reported that the Baltimore Dental College had adhered strictly to the regulations, both in the letter and the spirit; and had no cause for regret. The Faculty, Students, and all concerned are much pleased with the *regulations*, and gratified with their operation. The result thus far warrants their continuance.

The Dr. remarked that the adoption of these rules had prevented from 12 to 15 students attending the Baltimore College, who would otherwise have been there. There were 69 students. The examination of the candidates was thorough and rigid. Did not think a session of five months too long.

Prof. Kingsley remarked that the faculty of the New York

College of Dentistry, had proceeded in strict accordance with the regulations adopted by this Association.

The teaching in practical Dentistry had been largely of clinic or demonstrative character; that so far as operative Dentistry was concerned, very few didactic lectures had been given during the course; in the mechanical department, however, the instruction had been to a much greater extent, by lectures.

Assistants had been each day with the class in the work room. He gave a description of a book prepared for the purpose, by which the industry of the student may be known at all times. He also referred briefly to the mode of teaching employed by the various Professors of the Institution. At the close of Professor K's remarks, the Association adjourned till 2 o'clock, P. M.

AFTERNOON SESSION.

Association met pursuant to adjournment. President Parmley in the chair.

Minutes of the morning session were read and approved.

Prof. H. E. Peebles, of the Missouri Dental College, reported that the Institution which he represented, had conformed in every material point with the regulations adopted by this Association. He regards them as calculated to promote progress; thinks they should be strictly adhered to, and that other measures should be adopted so soon as found practicable.

Prof. Taft reported that the Ohio Dental College had adhered strictly to all the regulations of this Association, with one slight exception, which consisted in closing the session on the 4th of March, instead of the 15th, which, however, was not determined upon till near the close of the session. He referred to the circumstance which made it necessary to close the session at that time; they were of such a character as could not be disregarded. He further remarked, that the Ohio Dental College had adopted some additional regulations, which he regarded as very important, one of which is, a division of the studies into a junior and senior course. Another is, the possession of a good English education, as a requisite for entering the Institution. He referred somewhat in detail to the method of carrying out these regulations. A number of students had gone to other Institutions, who would have come to this, had the regulations been less stringent. Do not think the session too long.

Prof. R. King Browne, spoke at length on the general subject of teaching; referred particularly to the imperfect method of teaching Anatomy; thinks the teaching has been too much of a didactic character, there should be far more demonstration, it should accompany the lectures in all cases. The mere committing to memory the names, location, etc., of the various parts of the body, is not learning, but simply memorizing, which is an exceedingly ephemeral thing. The study of the tissues by actual minute examination is the true method of learning anatomy, the ordinary method of teaching will never make anatomists.

He regards the Dental profession as being in advance of any other profession in their efforts to improve their method of teaching.

Prof. Austen concurred fully in the views expressed by Dr. Browne. Disapproves most emphatically of the cramming process. In teaching anatomy, much improvement may be made in dissections and demonstrations, as well as in the didactic teaching. The ordinary method of dissection, fails in a large measure to accomplish the object aimed at; far more care should be exercised in this department, than is usual; would insist upon the closest attention to special anatomy; have frequently seen persons who supposed they had studied anatomy very thoroughly, in a little time, know nothing about it. He discussed the whole subject of Dental education at considerable length.

Upon invitation, Dr. W. H. Atkinson addressed the Association upon the subject of Dental education and Dental teaching. His remarks were very interesting, and were listened to with marked attention; he urged upon those engaged in teaching, the importance, aye, the necessity of occupying, and that immediately too, advance ground; and urged that they tarry not in the old ways. His remarks will not soon be forgotten by those who heard them.

The Association then adjourned till 10 o'clock, Friday morning.

SECOND DAY—MORNING SESSION.

Association met according to adjournment. President Parmley in the chair.

Minutes of the last session read and approved.

Prof. Gorgas inquired whether it was desirable to make any change in the regulations adopted at the last meeting of the Association.

Prof. Kingsley made some remarks, suggesting the propriety of shortening the session, taking the ground that to most students perhaps, as much instruction could be communicated in four, or four and a half months, if properly presented as in a longer term.

Prof. Taft opposed shortening the term; thinks five months short enough; is opposed to over working or cramming students; has usually found the session quite too short for the satisfactory performance of the work. Is decidedly opposed to letting down in the least upon any of the regulations heretofore adopted; prefers to see a still higher stand taken; he is certain the profession will sustain the colleges in taking a still higher position.

Prof. Peebles gave a somewhat detailed account of the organization of the Missouri Dental College. He expressed himself as decidedly opposed to making any change in the regulations, except in the direction of progress; thinks the length of term specified is not too long; would be glad to see higher positions taken. The Missouri Dental College will ever strive to be with the foremost, in elevating the standard of Dental education.

The following communications were now read by the Secretary.

PHILADELPHIA, April 1st, 1868.

*Prof. E. Parmley, President of the Association of the Colleges of Dentistry:*

DEAR SIR: I respectfully tender my resignation as Corresponding Secretary of the Association of the Colleges of Dentistry. Truly Yours,

J. H. MCQUILLEN.

PHILADELPHIA, April 1st, 1868.

*Prof. E. Parmley, President of the Association of the Colleges of Dentistry.*

DEAR SIR: At a meeting held on Wednesday, April 1st, 1868, It was—

*Resolved*, That the Faculty of the Philadelphia Dental College respectfully withdraw their connection with the Association of the Colleges of Dentistry.

*Resolved*, That the Dean be instructed to transmit this resolution to the above Association.

With respect, I remain Yours truly,

J. H. MCQUILLEN, *Dean.*

Prof. R. King Browne made some remarks upon the character of these communications. Thought it very unusual as well as discourteous, to send communications of this character and for this purpose, without having given any previous intimation, and without giving even the shadow of a reason for the course indicated; it would have been far more satisfactory, had there been some reasons given. Is this movement based upon a mere mercenary object in the matter of Dental teaching? Has fear of competition anything to do with it?

The President, Dr. Parmley, expressed himself as much annoyed and greatly disappointed by this movement of the Philadelphia Dental College; could regard it in no other light than as an insult to the Association.

On motion of Dr. R. King Browne,—

*Resolved*, That the resignations be both accepted.

It was on motion,—

*Resolved*, That the 4th section of the By-Laws be amended to read as follows:

That eight years of Dental practice, or a satisfactory examination upon Anatomy, Physiology, Inorganic Chemistry and Practical Dentistry, including a regular pupilage, will be regarded as equivalent to one course of lectures.

After a free expression of opinion upon the general subject of Dental teaching, the Association adjourned to meet at Niagara Falls, at the time of the meeting of the American Dental Association.

J. Taft, *Secretary.*

